# SIRT6 protein deacetylase interacts with MYH DNA glycosylase, APE1 endonuclease, and Rad9–Rad1–Hus1 checkpoint clamp

**DOI:** 10.1186/s12867-015-0041-9

**Published:** 2015-06-11

**Authors:** Bor-Jang Hwang, Jin Jin, Ying Gao, Guoli Shi, Amrita Madabushi, Austin Yan, Xin Guan, Michal Zalzman, Satoshi Nakajima, Li Lan, A-Lien Lu

**Affiliations:** Department of Biochemistry and Molecular Biology, University of Maryland School of Medicine, 108 North Greene Street, Baltimore, MD 21201 USA; University of Pittsburgh Cancer Institute, University of Pittsburgh School of Medicine, 5117 Centre Avenue, Pittsburgh, PA 15213 USA; School of Medicine, Tsinghua University, No.1 Tsinghua Yuan, Haidian District, Beijing, 100084 China; Department of Otorhinolaryngology-Head and Neck Surgery, University of Maryland School of Medicine, 16 South Eutaw Street, Baltimore, MD 21201 USA; Department of Microbiology and Molecular Genetics, University of Pittsburgh School of Medicine, 450 Technology Drive, Pittsburgh, PA 15219 USA; Marlene and Stewart Greenebaum Cancer Center, University of Maryland School of Medicine, 108 North Greene Street, Baltimore, MD 21201 USA; University of Maryland School of Nursing, 655 West Lombard Street, Baltimore, MD 21201 USA; Department of Natural and Physical Sciences, Life Sciences Institute, Baltimore City Community College, 801 West Baltimore Street, Baltimore, MD 21201 USA

**Keywords:** DNA repair, Sirtuin 6 (SIRT6), MutY homolog, APE1, Checkpoint clamp, Rad9/Rad1/Hus1, Telomeres, Protein–protein interaction

## Abstract

**Background:**

SIRT6, a member of the NAD^+^-dependent histone/protein deacetylase family, regulates genomic stability, metabolism, and lifespan. MYH glycosylase and APE1 are two base excision repair (BER) enzymes involved in mutation avoidance from oxidative DNA damage. Rad9–Rad1–Hus1 (9–1–1) checkpoint clamp promotes cell cycle checkpoint signaling and DNA repair. BER is coordinated with the checkpoint machinery and requires chromatin remodeling for efficient repair. SIRT6 is involved in DNA double-strand break repair and has been implicated in BER. Here we investigate the direct physical and functional interactions between SIRT6 and BER enzymes.

**Results:**

We show that SIRT6 interacts with and stimulates MYH glycosylase and APE1. In addition, SIRT6 interacts with the 9-1-1 checkpoint clamp. These interactions are enhanced following oxidative stress. The interdomain connector of MYH is important for interactions with SIRT6, APE1, and 9–1–1. Mutagenesis studies indicate that SIRT6, APE1, and Hus1 bind overlapping but different sequence motifs on MYH. However, there is no competition of APE1, Hus1, or SIRT6 binding to MYH. Rather, one MYH partner enhances the association of the other two partners to MYH. Moreover, APE1 and Hus1 act together to stabilize the MYH/SIRT6 complex. Within human cells, MYH and SIRT6 are efficiently recruited to confined oxidative DNA damage sites within transcriptionally active chromatin, but not within repressive chromatin. In addition, Myh foci induced by oxidative stress and Sirt6 depletion are frequently localized on mouse telomeres.

**Conclusions:**

Although SIRT6, APE1, and 9-1-1 bind to the interdomain connector of MYH, they do not compete for MYH association. Our findings indicate that SIRT6 forms a complex with MYH, APE1, and 9-1-1 to maintain genomic and telomeric integrity in mammalian cells.

**Electronic supplementary material:**

The online version of this article (doi:10.1186/s12867-015-0041-9) contains supplementary material, which is available to authorized users.

## Background

Reactive oxygen species and radiation cause DNA strand breaks and base lesions, thus affecting genomic integrity. Particularly, the C-G-rich telomeres are highly susceptible to oxidative damage [1, 2]. A frequent and highly mutagenic oxidative lesion is 8-oxo-7,8-dihydroguanine (G^o^), which mispairs with adenine during DNA replication, resulting in G:C to T:A mutations [3]. Oxidative DNA lesions are repaired primarily by the base excision repair (BER) pathway [4]. The first step in BER is carried out by a DNA glycosylase, which cleaves the damaged or mismatched base. The resulting apurinic/apyrimidinic (AP) site is processed by AP-endonuclease 1 (APE1), allowing the downstream BER enzymes to complete the DNA repair process [5].

MutY homolog (MYH or MUTYH) excises adenines from A/G^o^ mismatches and thus reduces G:C to T:A mutations [6–8]. Mutations in the human *MYH* (*hMYH*) gene can lead to colorectal cancer (as in MYH-associated polyposis or MAP) [9]. BER is coordinated with other cellular processes in eukaryotic cells [10]. Eukaryotic MYH contains unique motifs to mediate interactions with partner proteins involved in DNA replication, mismatch repair, and DNA damage response (reviewed in [6, 7]). We have shown that the interdomain connector (IDC, residues 295–350) located between the N- and C-terminal domains of hMYH is uniquely oriented [11] to interact with APE1 [12] and Hus1 [13], a subunit of the heterotrimeric Rad9–Rad1–Hus1 (9–1–1) checkpoint clamp. APE1 is essential for cell viability [14] and participates in many aspects of DNA metabolism and telomere maintenance [15–17]. In DNA repair, APE1 cleaves the phosphodiester bond 5′ to an AP site and removes various forms of 3′-blocked lesions at DNA strand breaks [5]. Because AP sites are mutagenic and cytotoxic [6], they must be recognized by APE1 immediately after the action of a DNA glycosylase. A “passing-the-baton” model has been proposed for BER [18], consistent with findings that APE1 stimulates many DNA glycosylases [19–23]. APE1 forms a stable complex with MYH and 9-1-1 [12, 24, 25]. Besides serving as a damage sensor, 9-1-1 is involved in many DNA metabolisms including BER (reviewed in [26]). Intriguingly, 9-1-1 interacts with and stimulates the activity of almost every enzyme in the BER pathway and has been proposed to serve as a platform to coordinate BER.

SIRT6 is a member of NAD^+^-dependent histone/protein deacetylase family (reviewed in [27]) and also has mono-ADP-ribosyltransferase and protein lysine fatty acyl removal activities [27, 28]. SIRT6 plays a role in stress response, DNA repair, telomere integrity, retrotransposition, and metabolic homeostasis [27, 29–31]. Sirt6 knockout mice display a shortened lifespan associated with impaired DNA repair [32]. Moreover, SIRT6 depletion leads to telomere dysfunction and premature cellular senescence [29, 30]. During the course of aging and in response to DNA damage, SIRT6 is depleted from L1 retrotransposon loci, allowing their activation [31]. SIRT6 has been implicated in BER [32]. It has been demonstrated that SIRT6 can activate PARP1 [33] and is a partner of thymine DNA glycosylase [34]. Xu et al. [35] recently reported that SIRT6 regulates BER in a PARP1-depdendent manner. However, direct physical and functional interactions between SIRT6 and BER enzymes remained to be elucidated. Here, we provide evidence for a direct role of SIRT6 in BER and DNA damage response (DDR). We show that mouse Sirt6 (mSirt6) interacts with MYH, APE1, and 9-1-1 and stimulates MYH and APE1 activities. Our data demonstrate that SIRT6, APE1, and Hus1 bind to hMYH without competition. Instead, one MYH partner enhances the association of the other two partners to the MYH complex. By using novel systems for confining oxidative DNA damage to defined human genomic regions, we show that MYH and SIRT6 are efficiently recruited to DNA damage sites within transcriptionally active chromatin, but not within inactive chromatin in human cells. In addition, we show that the number of Myh nuclear foci, frequently found to localize on telomeres, increase in *sirt6*^−/−^ mouse embryonic fibroblast cells. A further increase in telomere localization of Myh foci was found in the presence of oxidative stress. Thus, SIRT6 represents an interesting connection between chromatin remodeling and MYH-directed BER.

## Results

### SIRT6 interacts with MYH and APE1

**Figure 1:**
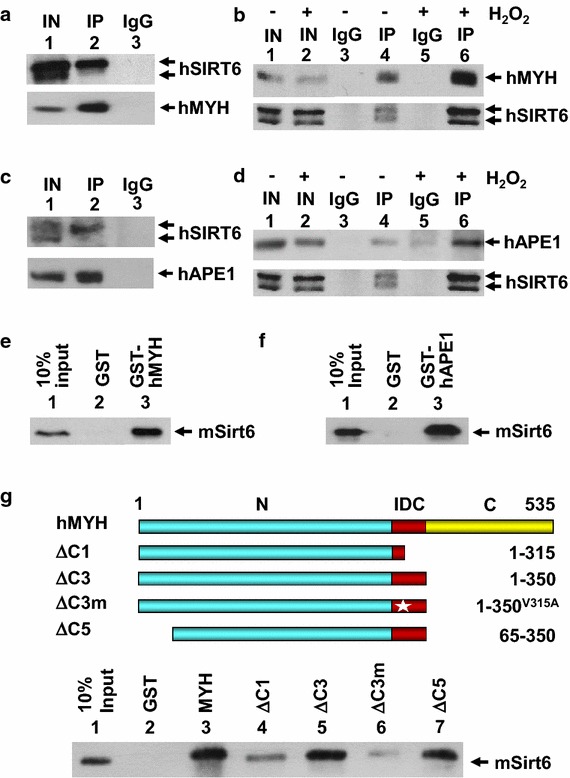
SIRT6 interacts with MYH, APE1, and Rad9–Rad1–Hus1. **a** SIRT6 can be co-immunoprecipitated by hMYH antibody from HeLa extracts. Immunoprecipitation (IP) was performed with hMYH antibody and detected on the Western blot using hSIRT6 or hMYH antibody (*lane 2*). *Lane 1* contains 10% of input cell extracts (IN). *Lane 3* is a negative control in which the immunoprecipitation was performed with IgG. **b** Interaction between hMYH and hSIRT6 is enhanced following H_2_O_2_ treatment. MYH was co-immunoprecipitated by SIRT6 antibody from extracts prepared from untreated HeLa cells (*lanes 4*) or from cells treated with 0.15 mM H_2_O_2_ for 1 h and recovered for 6 h (*lane 6*). Western blots were detected by hMYH or hSIRT6 antibody. Control lanes are similar to those described in (**a**). **c** SIRT6 and APE1 co-immunoprecipitated from HeLa extracts. Immunoprecipitation was performed with hAPE1 antibody and Western blotting was performed with hAPE1 or hSIRT6 antibody. Control lanes are similar to **a**. **d** Interaction between hAPE1 and hSIRT6 is enhanced following H_2_O_2_ treatment. APE1 was co-immunoprecipitated by SIRT6 antibody from extracts prepared from untreated (*lanes 4*) and H_2_O_2_ treated (*lane 6*) HeLa cells. Western blots were detected by hAPE1 or hSIR6 antibody. **e**, **f** Immobilized GST-hMYH (~1 μg) and GST-hAPE1 (~0.5 μg), respectively, were used to pull down FLAG-mSirt6 (0.1 μg). *Lane 1* contains 10% of input mSirt6 protein. *Lane 2* used GST-beads alone. FLAG-mSirt6 was detected by an anti-FLAG antibody. **g** Immobilized GST, GST-tagged intact hMYH, MYHΔC1, MYHΔC3, MYHΔC3m, and MYHΔC5 (shown in Additional file 1: Figure S2a) were used to precipitate FLAG-mSirt6. The hMYH constructs are depicted.

To examine whether SIRT6 plays a direct role in genomic integrity through BER, we analyzed the physical and functional interactions between SIRT6 and two major BER enzymes (MYH and APE1). First, association between SIRT6 and MYH was demonstrated by co-immunoprecipitation (Co-IP) (Figure 1a). Human cell extracts were subjected to immunoprecipitation with hMYH antibody followed by Western blot analysis with anti hMYH or hSIRT6 antibodies. Both hMYH and hSIRT6 were found in the pellet with hMYH antibody but not with control IgG. It is interesting to note that hMYH primarily interacts with the upper band of SIRT6 (Figure 1a, upper panel). We suspect the upper band may be a modified form of SIRT6 because SIRT6 can undergo auto mono-ADP-ribosylation [36]. Conversely, human cell extracts were subjected to immunoprecipitation with hSIRT6 antibody followed by Western analysis with hSIRT6 or hMYH antibodies. In this assay, both bands of hSIRT6 interacted with SIRT6 antibody. Human MYH was co-immunoprecipitated with hSIRT6 antibody (Figure 1b, upper panel). Using the similar approach, association between hSIRT6 and hAPE1 was established by co-IP (Figure 1c, d). As observed in hMYH-SIRT6 interaction, hAPE1 primarily interacts with the upper band of SIRT6 (Figure 1c, upper panel). Thus, hSIRT6 associates with hMYH and hAPE1.

To investigate the effect of DNA damage on the interactions between SIRT6 and BER enzymes, we performed Co-IP experiments with extracts from HeLa cells treated with 0.15 mM H_2_O_2_ for 1 h and recovered for 6 h. Interestingly, the interactions of hMYH and hAPE1 with hSIRT6 were enhanced after H_2_O_2_ treatment (Figure 1b, d, compare lanes 4 and 6). This result indicates that hSIRT6 interactions with BER enzymes are enhanced following oxidative treatment.

To show direct physical interactions between SIRT6 and BER enzymes, we performed GST-pull-down assays in the presence of ethidium bromide to eliminate the effect of nucleic acid on protein–protein interactions. Due to technical difficulties for purifying human enzymes, we purified mouse Sirt6 (mSirt6) and mouse MYH (mMyh) that is 83 and 77% identical to hSIRT6 and hMYH, respectively. This high conservation suggests that interactions between hMYH/mMyh1 and hSIRT6/mSirt6 may be interchangeable between human and mouse components. Both mSirt6 and mMyh were purified to more than 90% homogeneity as judged by coomassie blue staining and Western blotting (Additional file 1: Figure S1). Our data indicate that mSirt6 could be pulled down by GST-hMYH (Figure 1e). Similarly, interaction between mSirt6 and hAPE1 was established by GST-pull-down assays (Figure 1f). Thus, our results show that SIRT6 directly interacts with these two BER enzymes.

### The interdomain connector of MYH is important for interactions with SIRT6

To determine the regions of hMYH protein engaged in the physical interaction with mSirt6, we generated three hMYH deletion constructs fused to GST (Figure 1g). The purified proteins were analyzed by SDS–polyacrylamide gel electrophoresis as shown in Figure S2a in Additional file 1. Compared to intact MYH (Figure 1g, lane 3), only MYH(1–315) (ΔC1) had a reduced interaction with mSirt6 (Figure 1g, lane 4) while MYH(1–350) (ΔC3) and MYH(65–350) (ΔC5) had similar binding to mSirt6 (Figure 1g, lanes 5 and 7). Our results indicate that residues 316–350 of hMYH are critical for mSirt6-hMYH interaction. Interestingly, residues 295–350, constituting the interdomain connector (IDC) of hMYH [11], are also required for APE1 and Hus1 interactions [12, 13, 25]. We have shown that valine at position 315 (V^315^) of hMYH is important for Hus1 interaction but is dispensable for interaction with APE1 [13, 25]. To test whether V^315^ of hMYH is important for mSirt6 interaction, we analyzed the binding of mSirt6 with GST-tagged hMYH(1–350) containing a V315A mutation. The result (Figure 1g, compare lanes 5 and 6) demonstrates that the V315A mutant of hMYH substantially attenuated its interaction with mSirt6. Taken together, the IDC region of MYH is critical for association with Hus1, APE1, and SIRT6 and V^315^ of hMYH is important for SIRT6 and Hus1, but not for APE1, interactions.

### SIRT6 enhances the activities of MYH and APE1

**Figure 2:**
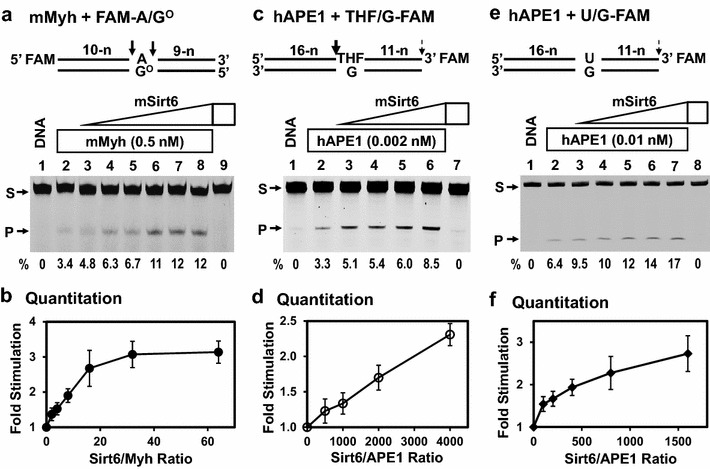
Sirt6 stimulates Myh and APE1 activities. **a** mSirt6 enhances mMyh glycosylase activity. DNA substrates used are shown with arrows indicating the cleavage sites. *Lane 1* 5′-FAM-labeled A/G^o^-containing DNA. *Lane 2* 5 nM A/G^o^-DNA was incubated with 0.5 nM mMyh. *Lanes 3–8* are similar to* lane 2* but with added 1, 2, 4, 8, 16, and 32 nM mSirt6, respectively. *Lane 9* A/G^o^-DNA was incubated with 32 nM mSirt6 without mMyh. *Arrows* mark the intact DNA substrate (S) and the cleavage product (P). Percentage (%) of product generated is shown below each* lane*. **c** mSirt6 enhances the AP endonuclease activity of hAPE1. *Lane 1* 3′-FAM-labeled tetrahydrofuran (THF)/G-DNA. *Lane 2* 20 nM THF/G-DNA was incubated with 0.002 nM hAPE1. *Lanes 3–6* are similar to* lane 2* but with added 1, 2, 4, and 8 nM mSirt6, respectively. *Lane 7* THF/G-DNA was incubated with 8 nM mSirt6 without APE1. **e** mSirt6 enhances the phosphodiesterase activity of hAPE1 that cleaves the 3′FAM from a U/G-containing DNA. *Lane 1* 3′-FAM-labeled U/G-DNA. *Lane 2* 20 nM U/G-DNA was incubated with 0.01 nM hAPE1. *Lanes 3–7* are similar to* lane 2* but with added 1, 2, 4, 8, and 16 nM mSirt6, respectively. *Lane 8* U/G-DNA was incubated with 16 nM mSirt6 without APE1. The product is a free FAM. **b**, **d**, and **f**, Quantitative analyses of the fold of stimulation from results as in **a**, **c**, and **e**, respectively. *Error bars* indicate SD; n = 3.

To determine the functional output of SIRT6 binding to MYH and APE1, we measured MYH and APE1 enzymatic activities in the presence of SIRT6. In these assays, we kept the ratios of mMyh and hAPE1 to DNA lower than 0.1 in order to observe better stimulation effect. Figure 2a shows that purified mSirt6 could enhance mMyh1 glycosylase activity on FAM-labeled A/G^o^-DNA. Quantification results (Figure 2b) showed that at a Sirt6/Myh ratio of 32, mSirt6 significantly enhanced mMyh activity by threefold (*p* ≤ 0.01). Human APE1 has very robust AP endonuclease activity and weak 3′-phosphodiesterase activity [37]. We observed that mSirt6 moderately stimulated both activities of hAPE1 (Figure 2c–f). Mouse Sirt6 stimulated the AP endonuclease activity of hAPE1 on FAM-labeled THF/G-DNA by twofold at a Sirt6/APE1 ratio of 3,000 (Figure 2c, d) (*p* = 0.001). Please note that the concentrations of APE1 and Sirt6 are 10,000 and 3.3 fold lower than the DNA concentration under this reaction condition. Because the weak 3′-phosphodiesterase activity of APE1 could not be detected using THF/G-DNA substrate, we used a U/G-containing DNA labeled with FAM at the 3′-end. In this case, APE1 does not cleave at the 5′ to the uracil, allowing the weak 3′-phosphodiesterase activity of APE1 to be detected. The phosphodiester bond between 3′-FAM and DNA could be cleaved by hAPE1 and this activity was enhanced by mSirt6 (Figure 2e, f). At a Sirt6/APE1 ratio of 400, mSirt6 could enhance the phosphodiesterase activity of hAPE1 by twofold (Figure 2e, f) (*p* ≤ 0.01). Under this reaction condition, the concentrations of APE1 and Sirt6 are 2,000 and fivefold lower than the DNA concentration. It is noteworthy that stimulation of MYH glycosylase activity by SIRT6 reaches saturation with increasing SIRT6 (Figure 2b), no saturation is observed for SIRT6 enhancement of the hAPE1 activities (Figure 2d, f) in the tested SIRT6/APE1 ratios. It may need higher SIRT6/APE1 ratios to reach saturation. As a comparison, it has been shown that hMYH can stimulate hAPE1 endonuclease activity by 2.7-fold with 10,000-fold molar excess of hMYH over hAPE1 [25]. Thus, Sirt6 stimulates enzymatic activities of hMYH and APE1 in vitro.

As shown above, both hMYH and hAPE1 primarily interacts with the upper band of SIRT6. Because SIRT6 can undergo auto mono-ADP-ribosylation [36], it is possible that the upper band of SIRT6 is a modified form of SIRT6. To determine whether mono-ADP-ribosyltransferase activity of SIRT6 is important for the stimulation of BER repair, we assayed SIRT6^G60A^ mutant which is defective in this activity [33]. SIRT6^G60A^ mutant had a significantly reduced ability to stimulate Myh glycosylase and APE1 endonuclease activities (Additional file 1: Figure S3) as compared to wild-type SIRT6, suggesting that mono-ADP-ribosyltransferase activity is important for their functional interactions.

### SIRT6 interacts with 9-1-1

**Figure 3:**
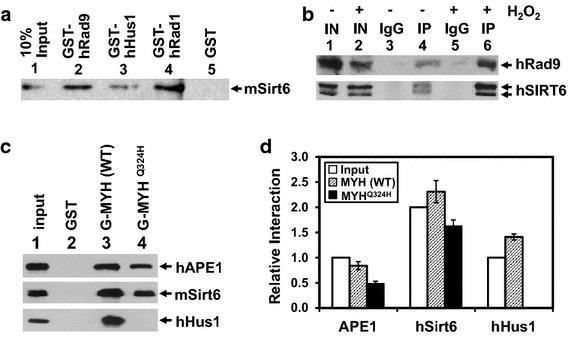
mSirt6 interacts with 9-1-1 and hMYH^Q324H^ does not interact with hHus1, but retains interaction with hAPE1 and mSirt6. **a** Immobilized GST, GST-tagged hRad9, hHus1, and hRad1 (shown in Additional file 1: Figure S2b) were used to pull-down FLAG-mSirt6. The procedures are similar to those described in Figure 1g.* Lane 1* contains 10% of input mSirt6 protein. **b** Interaction between hSIRT6 and hRad9 is enhanced following H_2_O_2_ treatment. Rad9 was co-immunoprecipitated by SIRT6 antibody from extracts prepared from untreated (*lanes 4*) and H_2_O_2_ treated (*lane 6*) HeLa cells. Western blots were detected by hRad9 or hSIRT6 antibody. *Lanes 1* and *2* contain 10% of input cell extracts (IN). *Lanes 3* and *5* are negative control in which the immunoprecipitation was performed with IgG. **c** Immobilized GST, GST-hMYH(1–350), and GST-hMYH(1–350)^Q324H^ (shown in Additional file 1: Figure S2C) were used to precipitate hAPE1, mSirt6, and hHus1.* Lanes 1* in* upper*,* middle*, and* lower panels* contain 10% of input hAPE1, 20% of input mSirt6, and 10% of input hHus1, respectively. Western blotting was performed with hAPE1, FLAG, or His antibodies. **d** Quantitative analyses of bound proteins on GST-tagged MYH constructs from three experiments. The Western blots were quantified by the ImageQuant Software (GE Healthcare). Relative interaction was calculated using input in *lane 1* as standard. *Open bars* input references, *stripped bars* bound proteins on GST-tagged wild-type MYH beads, *filled bars* bound proteins on GST-tagged mutant MYH beads.

Because MYH and APE1 interact with the 9-1-1 complex [11, 13, 24, 25], we tested whether SIRT6 had any interaction with the 9-1-1 complex. Equal molar of GST-tagged Hus1, Rad1, and Rad9 proteins (SDS–polyacrylamide gel shown in Additional file 1: Figure S2B) were separately immobilized on beads to pull down mSirt6. As shown in Figure 3a, mSirt6 bound strongly to GST-Rad1 (lane 4) and GST-Rad9 (lane 2), and weakly to GST-Hus1 (lane 3). Thus, Sirt6 binds to the 9-1-1 complex asymmetrically. SIRT6 binds weakly to the Hus1 subunit, while hMYH and hAPE1 bind preferentially to the Hus1 subunit [13, 24]. The unique structure of Hus1 may contribute to this asymmetry in protein–protein interactions. Association between hSIRT6 and hHus1 in vivo was established by co-IP (Figure 3b). The interaction of hSIRT6 with hRad9 was enhanced after H_2_O_2_ treatment (Figure 3b, compare lanes 4 and 6). Thus, hSIRT6 interactions with 9-1-1, MYH, and APE1 are all enhanced following oxidative stress. These results are consistent with a role of SIRT6 in DNA damage response [27, 38].

The hMYH^Q324H^ (or Q338H according to the new nomenclature) mutant found in MAP patients has been reported to attenuate its interaction with hHus1 and hRad9 by 80 and 50%, respectively, in comparison with wild-type hMYH [39]. To examine whether Q^324^ is important for SIRT6 and APE1 interaction, we analyzed the binding of mSirt6 and hAPE1 with GST-tagged hMYH(1–350)^Q324H^. We showed that GST-hMYH(1–350)^Q324H^ had no interaction with hHus1, but its interactions with hAPE1 and SIRT6 were only slightly reduced (Figure 3c, d). Thus, although Hus1, APE1, and SIRT6 bind to the IDC region of MYH, the binding is mediated by different sequence motifs.

### APE1, Hus1, and SIRT6 do not compete for MYH association

**Figure 4:**
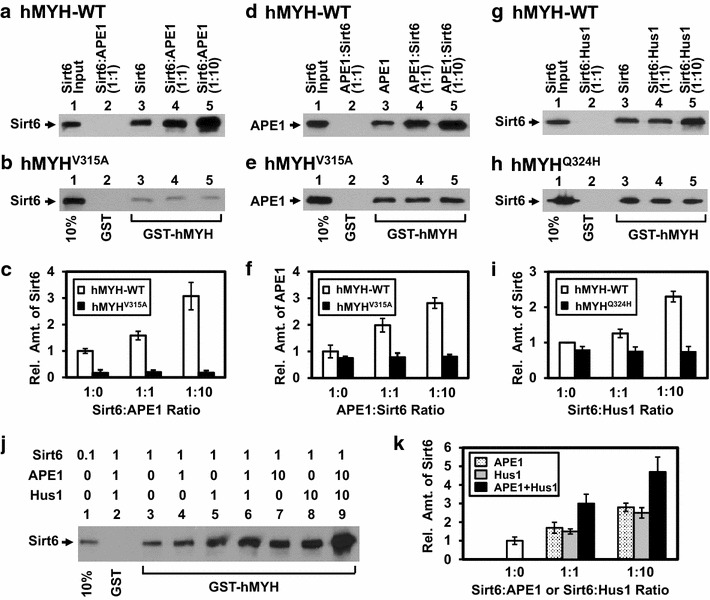
Association of SIRT6 with MYH is stabilized by APE1 and Hus1. **a**–**c** MYH/SIRT6 complex formation is enhanced by APE1. GST-hMYH(1–350)-WT (**a**) or GST-hMYH(1–350)^V315A^ (**b**) (shown in Additional file 1: Figure S2C) were used to pull-down mSirt6 in the presence of hAPE1. *Lanes 1* in both (**a**) and (**b**) contain 10% of input mSirt6 protein. *Lanes 2* in both (**a**) and (**b**) are GST alone. Western blots were detected by FLAG antibody. **c** Quantitation of the relative amount of Sirt6 in the precipitates from three experiments as in **a** and **b**. At an 1:1 (*lane 4*) and 1:10 (*lane 5*) molar ratio of mSirt6:hAPE1, the amount of mSirt6 pulled down by GST-hMYH-WT increases, but remains the same by GST-hMYH^V315A^. **d**–**f** MYH/APE1 complex formation is enhanced by SIRT6. Reactions are similar to **a**–**c**. GST-hMYH(1–350)-WT (**d**) or GST-hMYH(1–350)^V315A^ (**e**) (*lanes 3–5*) were used to pull-down hAPE1 in the presence of mSirt6. Western blots were detected by APE1 antibody. **g**–**i** MYH/SIRT6 complex formation is enhanced by Hus1. Reactions are similar to **a**–**c**. GST-hMYH(1–350)-WT (G) or GST-hMYH(1–350)^Q324H^ (**h**) (shown in Additional file 1: Figure S2C) (*lanes 3–5*) was used to pull-down mSirt6 in the presence of Hus1. Western blots were detected by FLAG antibody. **j** MYH/SIRT6 complex formation is enhanced by APE1 and Hus1. Reactions are similar to **a**. GST-hMYH(1–350)-WT was used to pull-down mSirt6 in the combination of APE1 and Hus1. Western blots were detected by FLAG antibody. **k** Quantitation of the relative amount of Sirt6 in the precipitates from three experiments as in **j**. The quantitation in **c**, **f**, **i**, and **k** is similar to that described for Figure 3c.

Next, we examined whether SIRT6, APE1, and Hus1 compete or stimulate each other for binding to MYH. We have shown that Hus1 enhances the MYH/APE1 complex formation [25]. Using the similar approaches, we performed GST pull-down assays of mSirt6 with immobilized GST-hMYH(1–350) (SDS–polyacrylamide gel of GST-hMYH shown in Additional file 1: Figure S2C) in the presence of increasing amounts of hAPE1 (Figure 4a). We observed that hAPE1 enhanced hMYH/mSirt6 interaction (Figure 4a, c, open bars). There is a threefold stimulation of mSirt6 binding to MYH when a tenfold excess of APE1 was added (Figure 4c, open bars). When we performed GST pull-down assays of mSirt6 with immobilized hMYH(1–350)^V315A^ mutant (SDS–polyacrylamide gel of GST-hMYH^V315A^ shown in Additional file 1: Figure S2C) in the presence of increasing amounts of hAPE1, neither enhancement nor inhibition was observed (Figure 4b, c, filled bars). Thus, the enhancement of the MYH/SIRT6 complex by APE1 requires a stable interaction between SIRT6 and MYH.

Inversely, we performed GST pull-down assays of hAPE1 with immobilized hMYH(1–350) in the presence of increasing amounts of mSirt6. mSirt6 also enhanced hMYH/hAPE1 interaction (Figure 4d, f, open bars). When we performed GST pull-down assays of hAPE1 with immobilized hMYH(1–350)^V315A^ mutant in the presence of increasing amounts of mSirt6, neither enhancement nor inhibition was observed (Figure 4e, f, filled bars). In another GST pull-down assay, hHus1 enhanced hMYH/mSirt6 interaction (Figure 4g, i, open bars) but did not enhance hMYH^Q324H^/mSirt6 interaction (Figure 4h, i, filled bars) (SDS–polyacrylamide gel of GST-hMYH^Q324H^ shown in Additional file 1: Figure S2C). Moreover, when APE1 and Hus1 were added to the pull-down assay of mSirt6 with immobilized hMYH(1–350), both stabilized the MYH/Sirt6 complex (Figure 4j, k). Taken together, our data suggest the formation of a functional DNA repair complex constituting of MYH, APE1, 9-1-1, and SIRT6. Our results also show that the enhancement of the MYH interaction with any one partner by a second protein partner requires a stable interaction between MYH and these proteins.

### Human MYH and SIRT6 are efficiently recruited to confined oxidative DNA damage within transcriptionally active chromatin, but not in inactive chromatin

**Figure 5:**
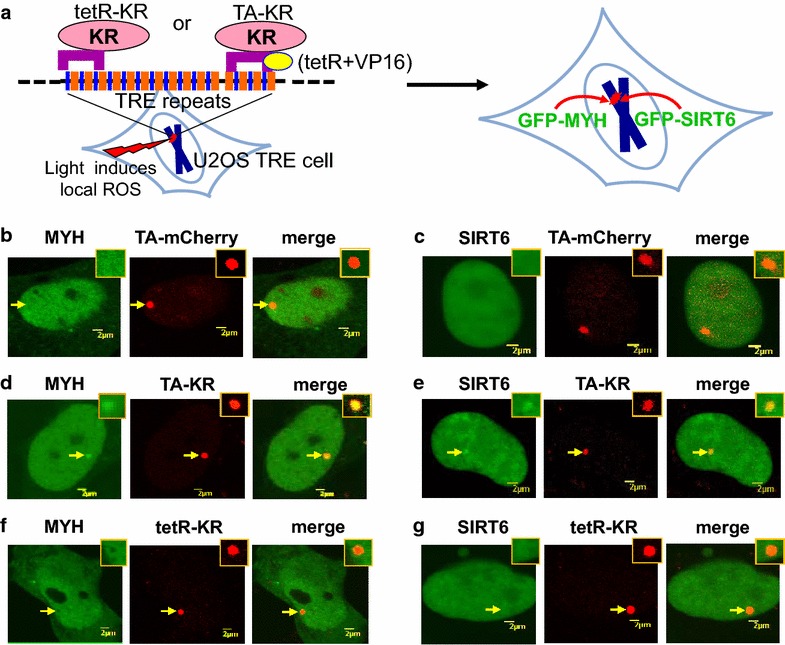
hMYH and hSIRT6 are recruited to oxidatively damaged sites located within transcriptionally active chromatin (TA-KR), but not within inactive chromatin (tetR-KR) in human U2OS TRE cells. **a** Scheme of tetR- and TA-tagged KR expression in the U2OS TRE cell [41]. To induce ROS-mediated damage at a specific locus in the human genome, we fused KR to the tetracycline repressor (tetR) to induce ROS damage in a 90-kb TRE array (total of 200 repeats) in U2OS cells. We also fused KR to the transcription activator tetR + VP16 (TA) to examine damage response at active chromatin. GFP-hMYH or GFP-hSIRT6 was co-transfected into the cells to analyze the recruitment of these proteins to the oxidative DNA damage sites. **b**, **c** GFP-MYH and GFP-SIRT6 are not enriched at sites with TA-mCherry in undamaged cells. **d**, **e** Damage response of GFP-MYH and GFP-SIRT6 to the site of TA-KR after light activation. **f**, **g** No recruitment of GFP-MYH and GFP-SIRT6 to the site of tetR-KR after light activation. Analyses of about 50 cells in each KR activated group indicated that over 90% of cells showed the colocalization of GFP-MYH foci or GFP-SIRT6 foci with TA-KR, in contract, none of the MYH or SIRT6 expressing cell showed foci at sites of tetR-KR.

It has been reported that SIRT6 responds to DNA double-strand breaks [33, 40] and SIRT6 might be important for the regulation of the chromatin states at the sites of damage. To investigate BER in vivo within chromatin, we have developed novel human systems for confining oxidative DNA damage to defined genomic regions within either transcriptionally inactive chromatin or active chromatin [41]. In this approach, local DNA damage is induced by activating site-specific KillerRed (KR) protein (a photosensitizer that generates ROS upon light irradiation) (Figure 5a). KR protein was fused to a tet-repressor (tetR-KR) or a transcription activator (TA-KR; TA = tetR + VP16) and was recruited to a defined genome site in human osteosarcoma U2OS cells via the interaction between tetR and integrated tetracycline responsive elements (TRE) (Figure 5a) [41]. Transcription is suppressed by tetR repressor alone but is activated in the presence of VP16. After activation of the KR by fluorescent light, local oxidative damage is induced within transcriptionally inactive (tetR-KR) or active (TA-KR) chromatin. Therefore, we applied the KR systems to examine the damage response of green fluorescence protein (GFP)-tagged MYH and GFP-SIRT6 following oxidative damage. Without DNA damage, GFP-MYH and GFP-SIRT6 were not enriched at sites with TA-mCherry, which serve as negative controls (Figure 5b, c). After light activation of the KR protein, both the frequency and intensity of 8-oxoG production and γH2AX foci at the sites of tetR-KR and TA-KR were similar [41], indicating that comparable amounts of DNA damage were produced by tetR-KR and TA-KR. Interestingly, GFP-tagged hMYH and hSIRT6 were only recruited to damage sites within transcriptionally active chromatin (TA-KR) (Figure 5d, e, yellow foci in the merged images), but not to damaged sites located within inactive chromatin (tetR-KR) (Figure 5f, g). For quantification, we analyzed 50 cells from each group. After KR activation, over 90% of cells expressing GFP-MYH or GFP-SIRT6 showed the colocalization of GFP-MYH foci or GFP-SIRT6 foci with TA-KR. In contrast, none of the GFP-MYH or GFP-SIRT6 expressing cell showed foci at sites of tetR-KR. Our data suggest that MYH and SIRT6 act together to repair oxidative DNA damages within transcriptionally active chromatin.

### Myh foci induced by oxidative stress and Sirt6 depletion are frequently localized on mouse telomeres

**Figure 6:**
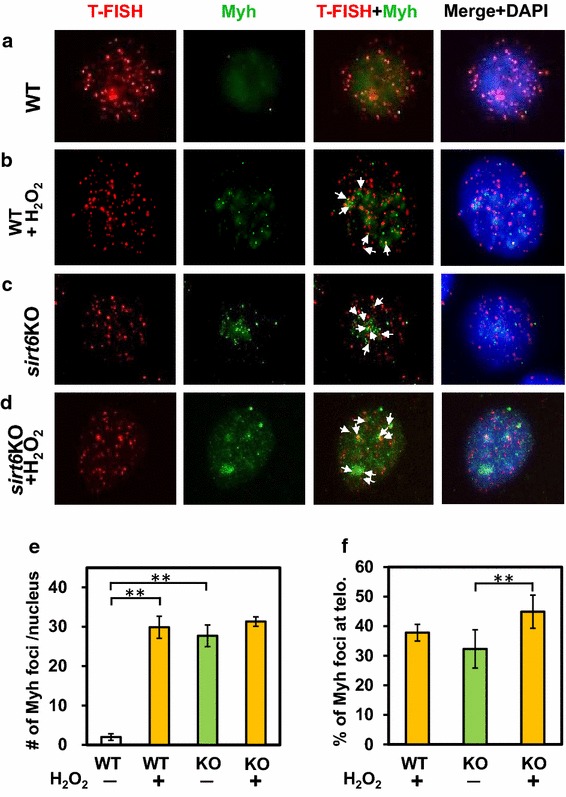
Myh foci induced by oxidative stress and Sirt6 depletion are frequently localized on telomeres. **a**, **b** Normal (WT) MEF cells; **c**, **d** *sirt6* ^−*/*−^ (knockout, KO) MEF cells. **b**, **d** Cells were treated with 0.3 mM H_2_O_2_ for 1 h and recovered for 4 h. Telomere FISH (T-FISH) in *red*, immunostained mMyh in *green*, DAPI-stained DNA in *blue*. *Arrows* indicate yellow mMyh foci localized on telomeres. **e** Quantitation of the number of mMyh foci per nucleus. **f** Quantitation of percentage of mMyh foci on telomeres [*yellow*/(*green* + *yellow*) in 3rd columns of (**b**–**d**)]. Experiments were repeated twice and more than 6 images were analyzed. Error bars indicate SD; n ≥ 6. The data of wild-type cell without oxidative stress is not included because the number of mMyh foci is very few [(**a**, **e**)].* Two stars *indicate that *p* values are smaller than 0.05.

Telomeres contain highly C:G rich repetitive DNA sequences and specific protein factors at the ends of chromosomes. These structures are highly susceptible to oxidative damage [2] and maintaining their integrity requires efficient BER [42–44]. It has been shown that human SIRT6, APE1, and 9-1-1 are associated with telomeres and are essential for telomere stability [17, 29, 45]. We have shown that *Schizosaccharomyces pombe* Myh1 is enriched on telomeres [46]. To examine whether mammalian MYH is co-localize at telomeres, we performed MYH immunostaining along with telomere fluorescence in situ hybridization (T-FISH) [29]. We compared normal (WT) and *sirt6* knockout (KO, *sirt6*^−*/*−^*)* mouse embryonic fibroblast (MEF) cells following oxidative DNA damage by H_2_O_2_ treatment. H_2_O_2_ treatment to *sirt6*^+/+^ (wild-type) cells substantially induced nuclear mMyh foci formation (Figure 6b, e) with 35% of Myh foci localized to telomeres (Figure 6b, f). Surprisingly, we observed that Myh foci increased in the *sirt6*^−/−^ cells (30% localized on telomeres) even without oxidative stress (Figure 6c, e, f). This may be consistent with a previous finding that SIRT6 knockdown leads to increased γ-H2AX foci at telomeres in human cells [29] indicating that deficiency of SIRT6 leads to telomere dysfunction. H_2_O_2_ treatment to *sirt6*^−/−^ cells slightly increased the number of mMyh foci (Figure 6e) with about 45% localized on telomeres (Figure 6d, f). These data suggest that MYH may play a role in repairing oxidative DNA damage at telomeres.

## Discussion

SIRT6 functions as an ADP-ribosyltransferase and protein deacetylase, and can remove the fatty acyl modification from proteins [27, 28], thus affecting many cellular functions including genomic stability. It has been shown that SIRT6 is involved in DNA double-strand break repair through interacting with several factors. SIRT6 promotes DNA double-strand break repair by interacting with DNA-dependent protein kinase (DNA-PK) and recruiting DNA-PK to chromatin at double-strand breaks [40]. SIRT6 mono-ADP-ribosylates PARP1 (a protein involved in both double-strand break repair and BER) and stimulates the poly-ADP-ribosyltransferase activity of PARP1 [33] while SIRT6 deacetylates CtIP [C-terminal binding protein (CtBP) interacting protein] [47]. In addition, SIRT6 recruits the chromatin remodeling factor SNF2H to double-strand breaks which, in turn is required for proper recruitment of downstream DDR factors and efficient DNA repair [38]. SIRT6 has been suggested to be involved in BER based on the phenotypes of *Sirt6* knockout mouse cells [32]. Three recent publications support SIRT6′s role in BER. Polyakova et al. [34] used yeast two-hybrid analyses to identify thymine DNA glycosylase as a partner of SIRT6; however, the authors did not further examine their direct physical and functional interactions. Mao et al. [33] reported that SIRT6 activates PARP1 and Xu et al. [35] recently reported that SIRT6 regulates BER in a PARP1-dependent manner. While those reports show the participation of SIRT6 in BER, our results provide a detailed mechanism of SIRT6 function in BER.

In this study, we provide the first evidence that SIRT6 interacts with MYH, APE1, and the 9-1-1 complex and these interactions are enhanced following oxidative stress. SIRT6 stimulates the enzymatic activities of MYH and APE1 in vitro in the absence of NAD^+^. These findings suggest that SIRT6 does not modify MYH or APE1 in our in vitro assays. Consistent with our observation, Xu et al. [35] stated that they did not identify BER proteins which could be deacetylated by SIRT6. However, it remains to be tested whether SIRT6 regulates these interacting partners in vivo through protein modification. Interestingly, the SIRT6-MYH interaction involves the inter-domain connector of hMYH that is also important for association with APE1 [12, 25] and Hus1 [13]. However, SIRT6, APE1, and Hus1 bind MYH through overlapping but different sequence motifs. The hMYH V315A mutation attenuates its interaction with hHus1 [13] and SIRT6 (Figure 1g) but not with hAPE1 [25] while the hMYH Q324H mutation abolishes its interaction with hHus1 [39] but not with hAPE1 and SIRT6 (Figure 3c). By GST pull-down assays in the presence of more than two MYH protein partners, we did not detect any competition between APE1, Hus1, or SIRT6 binding to MYH. Rather, one MYH partner enhances the association of the other two to MYH (Figure 4). These data suggest that SIRT6, Hus1, and APE1 may form a complex with MYH. How these three partner proteins interact with MYH within this short ~50-residue region remains to be determined. The structure of the IDC (residues 295–350) of hMYH has been shown to adopt a stabilized conformation projecting away from the catalytic domain [11] and may be suitable to form a docking scaffold for 9-1-1, APE1, and SIRT6.

The ability of Hus1 to stabilize the MYH/APE1/SIRT6 complex supports the model that 9-1-1 serves as a platform to coordinate BER [26] and maximize repair efficiency. We observed that the enhancement synergy of the MYH interaction with its partners is lost with hMYH^V315A^ and hMYH^Q324H^ mutants. hMYH^Q324H^ variant, found in MAP patients, is defective in interacting with 9-1-1 [39]. The phenotypes of hMYH^Q324H^ variant, as observed in *myh* knockout mouse embryo fibroblasts, are associated with increased G^o^ levels, hypersensitivity to oxidants, and accumulation of the cell population in the S phase of the cell cycle [39]. Some of these repair-defective phenotypes of hMYH^Q324H^ may be attributed by its inefficient recruitment of SIRT6 or APE1 to the damage sites.

Although SIRT6 physically interacts with MYH and APE1, mSirt6 stimulation on mMyh and hAPE1 activities is very subtle. At first glance, the effects of these functional interactions appear minor, as the glycosylase activity of mMyh increases only threefold in the presence of 30-fold excess of mSirt6. Even worse, the APE1 activities increase only twofold in the presence of 400-3,000-fold excess of mSirt6. However, these in vitro reactions were performed with enzyme concentrations far lower than DNA concentrations. It seems unlikely that SIRT6 would attain intracellular concentrations that would be 1,000-fold higher than APE1 in mammalian cells. Because AP endonuclease activity of hAPE1 is very robust, it may not require further stimulation by other factors. However, this mild stimulation on BER enzymes has been found in several cases. For example, we have shown that MYH can be enhanced by fourfold with tenfold molar excess of 9-1-1 [13] and can be stimulated by fourfold with 125-fold molar excess of APE1 [20, 23]. In addition, APE1 activity can be moderately stimulated by MYH with 10,000-fold molar excess of MYH over APE1 [25]. Although the interactions of MYH with SIRT6, APE1, and 9-1-1 produce modest stimulation on MYH catalytic activity in vitro, these interactions may be physiologically significant. We have demonstrated that a catalytically active SpMyh1^I261A/E262Q^ mutant, which corresponds to the hMYH^V315A/E316Q^ mutant, cannot reduce the mutation frequency of *myh1*Δ cells [11]. In addition, expression of a peptide consisting of the IDC of SpMyh1 that interferes with the interactions between SpMyh1 and interacting proteins in *S. pombe* cells makes cells more sensitive to H_2_O_2_ [11]. Such a regulatory network of weak protein interactions may offer the BER pathway sufficient flexibility to coordinate with DNA replication, DNA damage response, and other DNA repair pathways.

**Figure 7:**
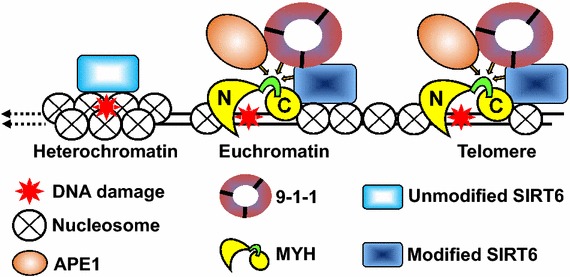
A model for SIRT6 interaction with MYH, APE1, and Rad9-Rad1-Hus1 to repair DNA damages on transcriptionally active chromatin and telomeres. The N- and C-terminal domains (in *yellow*) of MYH are connected with the interdomain connector (IDC, in *green*). SIRT6, APE1, and Rad9-Rad1-Hus1 bind overlapping but different sequence motifs on IDC region of MYH. The model suggests that SIRT6 in the complex with MYH, APE1, and 9-1-1 at sites of DNA damage may undergo auto mono-ADP-ribosylation leading to enhanced chromatin remodeling and optimal DNA repair efficiency.

Interestingly, MYH and APE1 prefer to bind to the upper band of SIRT6 in co-immunoprecipitation analyses. Although the nature of the upper band of SIRT6 is unknown, we suspect it may be a modified form of SIRT6. It has been shown that SIRT6 can undergo auto mono-ADP-ribosylation which may contribute to the self-regulation of SIRT6 function [36]. We also observed that mono-ADP-ribosylation defective SIRT6^G60A^ mutant could not efficiently stimulate Myh glycosylase and APE1 endonuclease activities as compared to wild-type SIRT6. This suggests that auto mono-ADP-ribosylation is important for SIRT6 function in BER. Thus, we suggested that the modified form of SIRT6 participates in BER. We favor a model that SIRT6 in the complex with MYH, APE1, and 9-1-1 at sites of DNA damage may undergo auto mono-ADP-ribosylation leading to enhanced chromatin remodeling and optimal DNA repair efficiency (Figure 7).

hMYH and hSIRT6 are efficiently recruited to KR-induced confined oxidative DNA damage within transcriptionally active chromatin, but not the DNA damage within inactive chromatin. This property of hMYH is unique because other DNA glycosylases such as NTH1, NEIL1, NEIL2, and MBD4 are recruited to both transcriptionally active chromatin and inactive chromatin [41]. The preferred recruitment at sites of DNA damage within active chromatin is also found with FEN1 and PCNA [41], consistant with the reports that MYH interacts with PCNA in the long-patch BER pathway [12]. However, the determinants for long-patch BER pathway occurs at transcriptionally active chromatin are not clear. Because MYH needs to recognize both the mismatched adenine and G^o^, transcriptionally inactive heterochromatin may be not accessible to MYH even in the presence of SIRT6. SIRT6 has been shown to interact with PARP1 [33] and to regulate BER in a PARP1-depdendent manner [35], however, PARP1 is recruited to DNA damage within transcriptionally inactive chromatin more efficiently than in active chromatin [41]. Although this differential response to KR-induced oxidative damage is not fully understood, this may imply that SIRT6 stimulation of PARP1 activity at TA-KR-bound sites may be subtle and that PARP1 may play minimal role in MYH-mediated long-patch BER. The PARP1-depdendent BER analyzed by plasmid reactivation assay used by Xu et al. [35] may not involve MYH.

Telomeric DNA are highly susceptible to oxidative damage compared to other regions of the chromatin [2]. Oxidative damage to telomeric DNA accelerates telomere shortening and requires efficient DNA repair to maintain its integrity [42–44]. In addition, chromatin at telomeres contains hypoacetylated histones [48] and requires remodeling to give the repair machinery full access to sites of DNA damage. It has been shown that human SIRT6, APE1, and 9-1-1 are associated with telomeres and are essential for telomere stability [17, 29, 45]. Similar to *S. pombe* Myh1 [46], for the first time, we show that mouse Myh is associated with damaged telomeres. It has been shown that γ-H2AX foci increase at telomeres in SIRT6 knockdown cells [29]. This telomere dysfunction is consistent with increased MYH foci at telomeres in *sirt6* knockout cells without oxidative damage (Figure 6c). The interaction of 9-1-1 with SIRT6 and BER enzymes ensures that DNA repair, chromatin remodeling, and DDR are coordinated. Our findings indicate that SIRT6 interacts with MYH, APE1, and 9-1-1 to maintain genomic integrity of telomeres and transcriptionally active chromatin, but not transcriptionally inactive chromatin (Figure 7). Our model suggests that SIRT6 in the complex with MYH, APE1, and 9-1-1 at sites of DNA damage may undergo auto mono-ADP-ribosylation leading to enhanced chromatin remodeling and optimal DNA repair efficiency. The similar response of SIRT6 and MYH to oxidative DNA damage within transcriptionally active chromatin suggests that SIRT6 may alter the chromatin structure and facilitate DNA repair. Because MYH needs to recognize both the mismatched adenine and the G^o^ located on the other strand, the DNA glycosylase activity of MYH is expected to be strongly prohibited on nucleosome-bound mismatches. Thus, MYH repair may require substantial nucleosome remodeling to expose the mismatch for efficient repair. It has been reported that SIRT6 recruits the chromatin remodeler SNF2H to double-strand breaks and focally deacetylates histone H3K56 [38]. It will be interesting to see whether SIRT6 recruits SNF2H to oxidatively damaged telomeres to enhance BER.

## Conclusions

The results presented in this study demonstrate that SIRT6 has a direct role in BER by forming a complex with and stimulating MYH and APE1. Our finding that SIRT6 interacts with the 9-1-1 complex suggests SIRT6 may have a role in DDR and is consistent with the finding that the lack of SIRT6 profoundly impacts upon downstream recruitment of DNA repair factors [38]. Our data demonstrate that SIRT6, APE1, and Hus1 bind to the IDC region of hMYH without competition. We demonstrate that hMYH and hSIRT6 are efficiently recruited to confined oxidative DNA damage in transcriptionally active chromatin in human cells and that mMyh foci induced by oxidative stress and mSirt6 depletion are frequently localized on mouse telomeres. Overall, our findings suggest that SIRT6 forms a complex with MYH, APE1, and 9-1-1 to maintain genomic and telomeric integrity. SIRT6 interactions with MYH, APE1, and 9-1-1 fit well with the observed marked genomic instability of SIRT6 deficient cells and aging phenotypes of *sirt6* knockout mice [29, 30, 32]. Taken together, our data highlight a potential role of chromatin remodeling in DNA repair and DDR.

## Methods

### Glutathione-S-transferase (GST) fusion protein constructs

Full length cDNA of *hAPE1* was amplified by PCR using pET28-hAPE1 plasmid (a gift from Dr. Alex Drohat, University of Maryland Medical School) as template and the primers listed in Additional file 1: Table S1. The PCR product was digested with BamHI and XhoI and ligated into the digested pGEX-4T-2 (GE Healthcare) to yield the pGEX-hAPE1 construct.

Plasmids containing GST-hMYH, GST-hMYH(1–315), GST-hMYH(1–350), GST-hMYH(1–350)^V315A^, GST-Rad9, GST-Rad1, and GST-Hus1 have been described by Shi et al. [13]. The plasmid containing GST-hMYH(65–350) was derived from pET19b-hMYH(65–350) [11] by PCR amplification using primers listed in Additional file 1: Table S1. The PCR product was digested with BamHI and XhoI and ligated into the digested pGEX-4T-2 (GE Healthcare). The Gln^324^ to His (Q324H) mutant of the *hMYH* gene was constructed with the QuickChange site-directed mutagenesis kit (Stratagene) using the pGEX4T-hMYH(1–350) plasmid [13] as template and primers listed in Additional file 1: Table S1. The mutation was verified by DNA sequencing.

### Purification of mouse Sirt6 proteins

Full length mouse *Sirt6* cDNAs encoding wild-type and G60A mutant proteins cloned in pcDNA3.1 vector were gifts from Dr. Raul Mostoslavsky at Harvard Medical School.

Mouse *Sirt6* clones were transfected into the HEK293T human cells using X-tremegene HP reagents (Roche). Wild-type and G60A FLAG-tagged mSirt6 proteins were purified by affinity chromatography as described for FLAG-hSIRT1 [49]. The fractions that contain the FLAG-mSirt6 proteins (confirmed by SDS–polyacrylamide gel analysis and Western blotting) were pooled, divided into small aliquots, and stored at −80°C. The FLAG-mSirt6 proteins were ~90% pure (Additional file 1: Figure S1) and their concentrations were determined by SDS-PAGE and compared to bovine serum albumin standards.

### Purification of mouse Myh protein

Full length cDNA of *mMyh* cloned in pcDNA3.1 (kindly provided by Dr. Yusaku Nakabeppu at Kyushu University, Japan) [50] was subcloned into pET21a (EMD Bioscience) by PCR amplification using primers listed in Additional file 1: Table S1. The PCR product was digested with NheI and XhoI and ligated into the digested pET21a. Mouse Myh was purified similarly as described for hMYH [11] and was >90% pure (Additional file 1: Figure S1). There was one major degradation product (~38 kDa) of mMyh as judged by Western blotting.

### Other proteins used

APE1 was purified from BL21(DE3) cells (Novagen) containing pET28-hAPE1 as published [51]. His-tagged hHus1 was purified from BL21-Star cells (Novagen) containing pET21a-hHus1 [13].

### Cell culture and cell extracts

Human HeLa S3 and HEK-293T cell lines were purchased from American Type Culture Collection (ATCC). HeLa cells were maintained in DMEM (Cellgro) supplemented with 10% fetal bovine serum (FBS) and penicillin–streptomycin at 37°C in 5% CO_2_. HeLa cell extracts were prepared from cells grown to late log phase as described [52] or from cells treated with 0.15 mM H_2_O_2_ for 1 h and recovered for 6 h. HEK-293T cells were maintained in MEM (Invitrogen) supplemented with 10% fetal bovine serum and 1% Penicillin–Streptomycin. *Sirt6*^+/+^ (wild type, WT) and *sirt6*^−/−^ (knockout, KO) mouse embryonic fibroblast (MEF) cells (obtained from Dr. Raul Mostoslavsky at Harvard Medical School) were maintained in DMEM (Invitrogen) supplemented with 15% fetal bovine serum and 1% Penicillin–Streptomycin. MEF cells were treated with 0.3 mM H_2_O_2_ for 1 h and recovered for 4 h. U2OS TRE (also called as U2OS-SCE 19) cell line containing 200 copies of pTRE/I-SceI has been described [41]. U2OS cells were cultured in DMEM with 10% FBS at 37°C and transfected with plasmids with Fugene-6 (Life Technology).

### Antibodies and western blotting

The hMYH polyclonal antibodies (α344) against peptide residues 344–361 (FPRKASRKPPREESSATC) were raised in rabbits, purified as described [53], and were shown to cross-react with mMyh (unpublished data). The mMyh polyclonal antibodies against full-length mMyh used for immunostaining were raised in rabbits with Custom Antibody Service by Alpha Diagnostic International Inc. and purified as described [54]. Commercial antibodies used for Western blotting include: hSIRT6 (Cell Signaling), mSirt6 (Abcam), hAPE1 (Abcam), hRad9 (Imgenex), FLAG-tag (Sigma-Aldrich), His-tag (Santa Cruz Biotechnology), β-actin (Sigma-Aldrich), and horseradish peroxidase-conjugated anti-mouse/anti-rabbit antibodies (BioRad). Western blotting was performed as described [52] and detected by the Enhanced Chemiluminescence (ECL) analysis system (USB Corporation, 72552) according to the manufacturer’s protocols.

### GST-pull-down and co-immunoprecipitation assays

The assays were performed similarly as described previously [25]. To eliminate the effect of nucleic acid on protein–protein interactions, 50 μg/ml of ethidium bromide was added to the immobilized proteins for 30 min prior adding their interacting partners.

### mMyh glycosylase activity assay

The Myh substrate is a 20-mer duplex DNA containing an A/8-oxoG (A/G^o^) base/base mismatch with the 5′end of the A-containing strand labeled with fluorescein (FAM) (Additional file 1: Table S1). The mMyh glycosylase assay was performed similarly as described [11]. The A/G^o^-DNA substrate (5 nM) was incubated with 0.5 Nm mMyh and different amounts of FLAG-mSirt6 at 37°C for 30 min. The products containing AP sites were then treated with 0.1 M NaOH at 90°C for 30 min to cleave the phosphodiester bonds. Reaction mixtures were loaded onto 14% sequencing gels containing 7 M urea. Images were detected with the Typhoon FLA9500 and quantified by the ImageQuant Software (GE Healthcare).

### APE1 activity assay

Two types of DNA substrates were used to assay hAPE1 activity. A 28-mer synthetic nucleotide (Additional file 1: Table S1) containing a single tetrahydrofuran (THF, an AP analog) and 3′ FAM was annealed with the complementary oligonucleotide with G opposing THF. The other APE1 substrate is a DNA duplex (28-mer) containing a U/G mismatch with 3′FAM labeled on the U-containing strand. The AP endonuclease assay mixture (10 μl) contained 50 mM Tris–HCl, pH 7.5, 5 mM MgCl_2_, 0.1 mg/ml BSA, 1 mM DTT, 10% glycerol and 20 nM 3′-FAM-labeled DNA. The reaction was preceded by adding either 0.002 nM (for THF/D-DNA) or 0.01 nM (for U/G-DNA) of hAPE1 and different amounts of FLAG-mSirt6 at 30°C for 30 min. Reaction mixtures were analyzed and detected similar as in mMyh glycosylase activity assay.

### KR activation to induce oxidative DNA damage

U2OS TRE cells were transiently transfected with plasmids described below. TA-mCherry, TA-KR, and TetR-KR plasmids have been described [41]. Full length cDNAs of *hMYH and hSIRT6* were subcloned by PCR amplification using primers listed in Additional file 1: Table S1. The PCR product of *hMYH* was digested with BamHI and XhoI and ligated into the BglII-SalI digested pEGFP-C1 vector (Clonetech Laboratories) while the PCR product of *hSIRT6* was digested with XhoI and NotI and cloned into the digested pEGFP-C1. 24 h after transfection, cells were exposed to a 15-W SYLVANIA cool white fluorescent bulb for 10 min in a stage UVP (Uvland) as described [41]. Images were captured 10 min after light activation with an Olympus FV1000 confocal microscopy system and FV1000 software.

### Immuno-telomere FISH assay

Cells were stripped from plates and treated with 75 mM KCl solution at 37°C for 6 min. After centrifugation, the cells were fixed with ice-cold methanol/acetic acid (3:1). The fixed cells were dropped to a slide on the floor with a ~20° angle from the distance more than 140 cm. After air-drying in a fume hood, the slides were pretreated with 3.7% formaldehyde and unmasking solution (ProHisto, LLC). Cells were then dehydrated sequentially with 70, 85, and 95% ethanol solution. Alexa Fluor-546-conjugated (CCCTAA)_3_ or (TTAGGG)_3_ probes (IDT) was then added to slides and heated in 87°C oven for 10 min and cooled down to room temperature for 1 h. After being blocked, cells were immunostained with mMyh antibody, followed by Alexa Fluor-488-conjugated anti-rabbit IgG (H+L) antibody (Molecular Probes). The immunostained cells were then counterstained with DAPI and mounted with coverslips. Images were captured with a Nikon PCM 2000 confocal microscope scanning system.

## Additional file

Additinal file 1: In the supplement Material Section results from the protein purification and respective SDS-PAGE as well as the data from the stimulation of a SIRT6 mutant on MYH and APE1 activities are presented.

## Abbreviations

9-1-1: Rad9, Rad1, and Hus1 heterotrimer complex
AP: apurinic/apyrimidinic
APE1: AP endonuclease
BER: base excision repair
DDR: DNA damage response
ECL: enhanced chemiluminescence
FAM: 6-carboxyfluorescein
G^o^ or 8-oxoG: 7,8-dihydro-8-oxo-guanine
GFP: green fluorescence protein
GST: glutathione-S-transferase
h: human
IDC: interdomain connector
KO: knockout
KR: KillerRed
m: mouse
MAP: MYH-associated polyposis
mCherry: a monomer red fluorescent protein
MEF: mouse embryonic fibroblast
MYH, MUTYH, or Myh: MutY homolog
NT: non-target
IP: pellet
PCR: polymerase chain reaction
ROS: reactive oxygen species
TA: transcription activator
tetR: tet-repressor
T-FISH: telomere fluorescence in situ hybridization
THF: tetrahydrofuran abasic site analog
TRE: tetracycline responsive elements
WT: wild-type
