# Supplementary material for: SIRT6 protein deacetylase interacts with MYH DNA glycosylase, APE1 endonuclease, and Rad9–Rad1–Hus1 checkpoint clamp
Source: BMC Mol Biol. 2015 Jun 11;16:12. doi: 10.1186/s12867-015-0041-9 (PMC4464616; doi:10.1186/s12867-015-0041-9)
Supplement: Additinal file 1: — In the supplement Material Section results from the protein purification and respective SDS-PAGE as well as the data from the stimulation of a SIRT6 mutant on MYH and APE1 activities are presented. [file 12867_2015_41_MOESM1_ESM.pdf]

SIRT6 Protein Deacetylase Interacts with MYH DNA Glycosylase, APE1 Endonuclease, and Rad9-Rad1-Hus1 Checkpoint Clamp

Bor-Jang Hwang[1], Jin Jin[1], Ying Gao[2,3], Guoli Shi[1,Φ], Amrita Madabushi[1,Φ], Austin Yan[1], Xin Guan[1], Michal Zalzman[1,4], Satoshi Nakajima[2,5], Li Lan[2,5], and A-Lien Lu[1,6,*]

[1] *Department of Biochemistry and Molecular Biology, University of Maryland School of Medicine, 108 North Greene Street, Baltimore, MD 21201, USA*

[2] *University of Pittsburgh Cancer Institute, University of Pittsburgh School of Medicine, 5117 Centre Avenue, Pittsburgh, Pennsylvania 15213, USA*

[3] *School of Medicine, Tsinghua University, No.1 Tsinghua Yuan, Haidian District, Beijing 100084, China*

[4] *Department of Otorhinolaryngology-Head and Neck Surgery, University of Maryland School of Medicine, 16 South Eutaw Street, Baltimore, MD 21201, USA*

[5] *Department of Microbiology and Molecular Genetics, University of Pittsburgh School of Medicine, 450 Technology Drive, Pittsburgh, Pennsylvania 15219, USA*

[6] *Marlene and Stewart Greenebaum Cancer Center, University of Maryland School of Medicine, 108 North Greene Street, Baltimore, MD 21201, USA*

Supplemental information

1. Table 1. Oligonucleotides used

Name	Sequence	Purpose
A-20	FAM-CCGAGGAATTAGCCTTCTGC	Myh1 substrate
G°-20	GCAGAAGGC(G°)AATTCCTCGG	Myh1 substrate
THF-28	GTGTCACCACTGCTCA(THF)GTACAGAGCTG-FAM	APE1 substrate
U-28	GTGTCACCACTGCTCAUGTACAGAGCTG-FAM	APE1 substrate
G28	CAGCTCTGTACGTGAGCAGTGGTGACAC	APE1 substrate
GST-hAPE1-For	TAAGGATCCATGCCGAAGCGTGG	hAPE1 5' primer
GST-hAPE1-Rev	CGCGCTCGAGCAGTGCTAGGTATA	hAPE1 3' primer
GST-hMYH(65-350)-F	GGCAGCGGATCCGCCTCTGTCTCCTCATACC	hMYH 5' primer
GST-hMYH(65-350)-R	GACTCGCTCGAGTCACTTGCGGCTGGCCTTTCTGC	hMYH 3' primer
mMyh-His-For	GATTTAGCTAGCATGAAGAAACTCCAAGCATC	mMyh 5' primer
mMyh-His-Rev	GATTTACTCGAGCTGGGTAGTACTGTTGGGTT	mMyh 3' primer
hMYH^{Q324H}-sense	GTGCTCCCAACACTGGACATTGCCACCTGT	hMYH mutagenesis primer
hMYH^{Q324H}-antisense	ACAGGTGGCAATGTCCAGTGTTGGGAGCAC	hMYH mutagenesis primer
GFP-hMYH	TATTAGGATCCATGACACCGCTCGTCTC	hMYH 5' primer
GFP-hMYH	TATTCTCGAGTTCACTGGGCTGCACTGT	hMYH 3' primer
GFP-hSIRT6	CTCGAGTCGGTGAATTACGCGGCGGGGCTGTCG	hSIRT6 5' primer
GFP-hSIRT6	GCGGCCGCTGCTGGGGACCGCCTTGGCCTTCAC	hSIRT6 3' primer

[a] The base which mismatches with the complementary strand is underlined.

2. Figure S1.

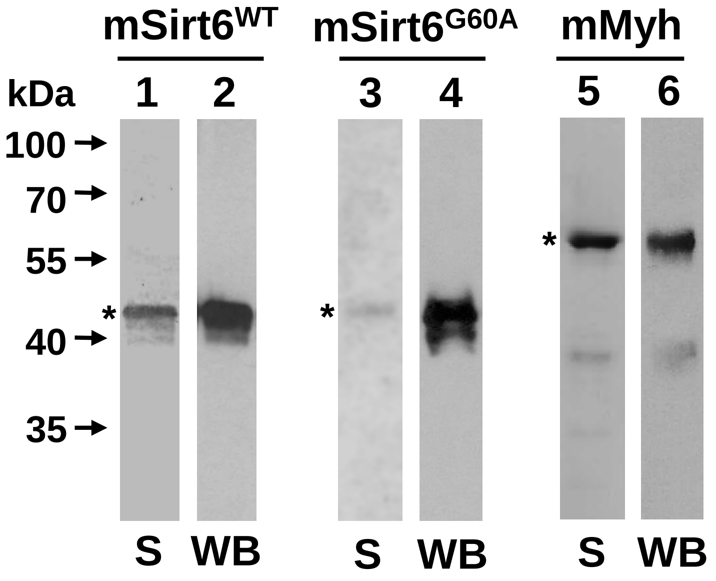

Figure S1. Purified FLAG-tagged mouse Sirt6 (mSirt6) and His-tagged mouse Myh (hMyh). FLAG-tagged mSirt6 was expressed in HEK-293T cells and purified by M2 affinity chromatography. His-mMyh expressed in *E. coli* was purified by nickel-sepharose, Q-sepharose, and heparin column. The proteins were fractionated on a 10% polyacrylamide gel in the presence of SDS. Lanes 1 and 2 contain FLAG-mSirt6^{WT}, lanes 3 and 4 contain FLAG-mSirt6^{G60A}, and lanes 5 and 6 contain His-mMyh. Odd lanes were stained with Coomassie Blue (S) and even lanes were from Western blots (WB) using anti-FLAG or anti-His antibodies. There are multiple protein bands and the stars mark the major bands of FLAG-mSirt6 and His-mMyh proteins. The positions of protein markers are indicated by arrows.

3. Figure S2

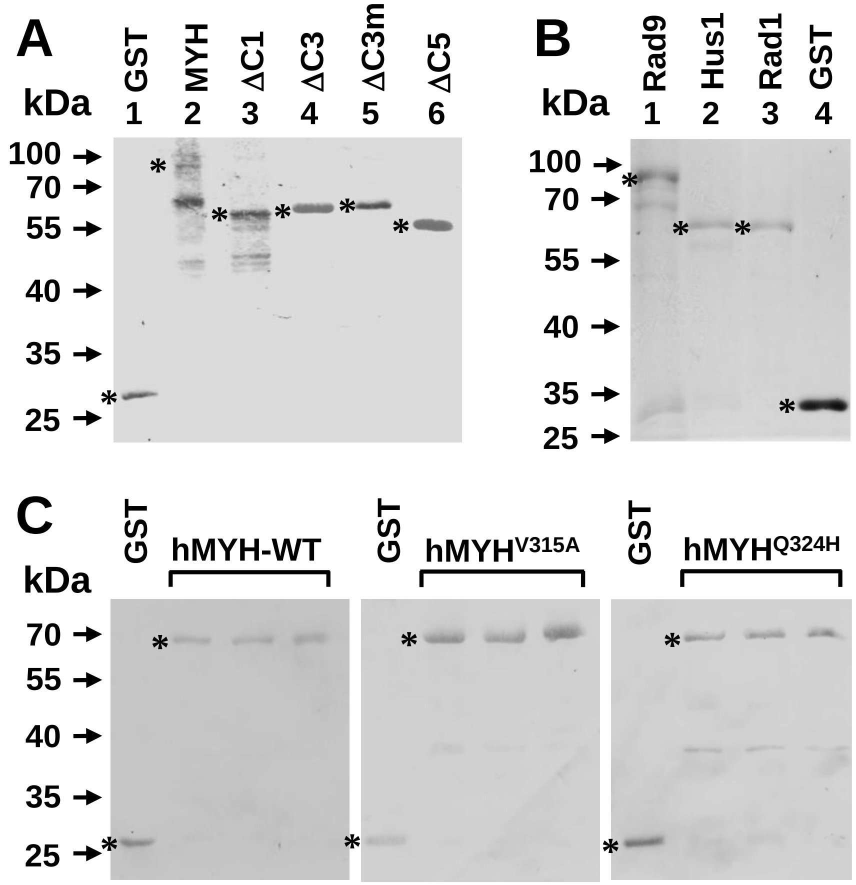

Fig. S2. SDS-polyacrylamide analyses of GST-tagged proteins used in the pull-down experiments. (**A**) GST-hMYH constructs used in Figure 1G. (B) GST-tagged hRad9, hHus1, and hRad1 used in Figure 3A. (**C**) GST-hMYH(1-350), GST-hMYH(1-350)^{V315A}, and GST-hMYH(1-350)^{Q324H} used in Figure 4. All fusion proteins (marked by stars) in gel or membrane were stained with Coomassie Blue. The positions of molecular weight markers are indicated

4. Figure S3

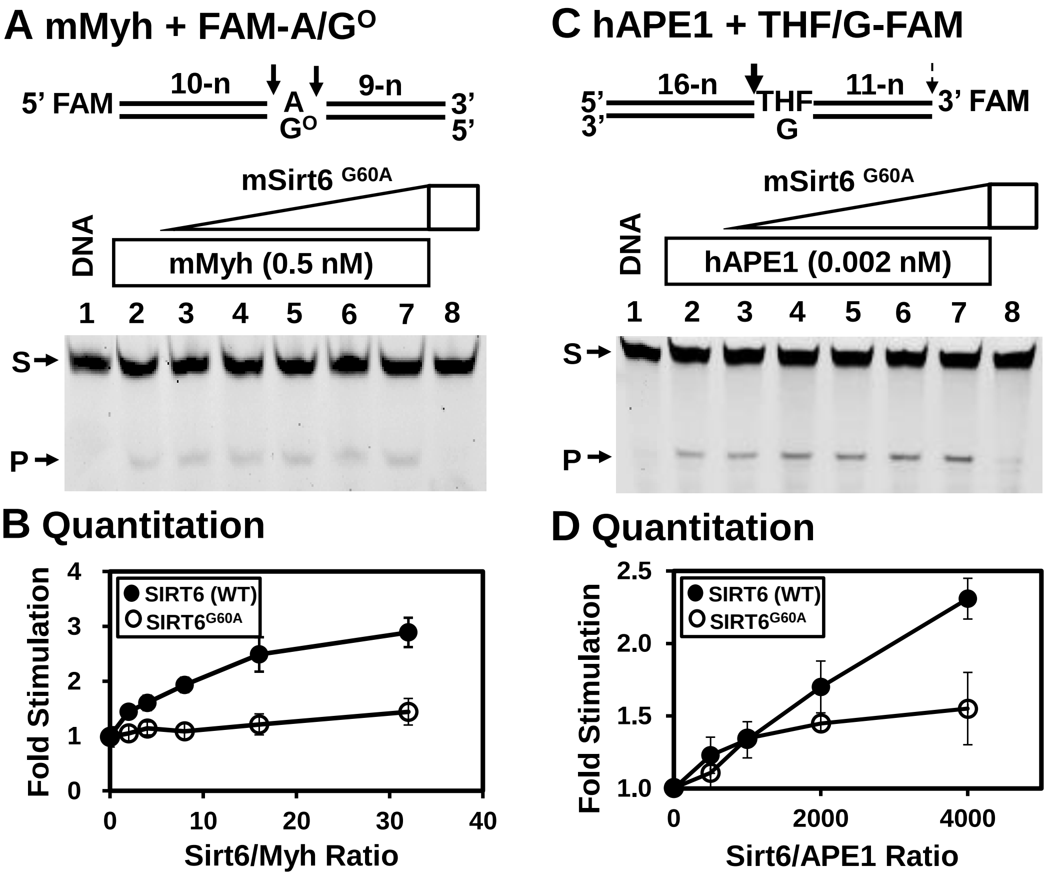

Figure S3. Sirt6^{G60A} stimulates Myh and APE1 activities weaker than wild-type SIRT6. **(A)** Sirt6^{G60A} enhances mMyh glycosylase activity. **(C)** Sirt6^{G60A} enhances the AP endonuclease activity of hAPE1. **(B)** and **(D)**, Quantitative analyses of the fold of stimulation of Sirt6^{G60A} as compared to wild-type SIRT6. Error bars indicate SD; n = 3. Experiments were performed similarly to those described in Figure 2.
